# Cannabidiol Loaded Topical Ophthalmic Nanoemulsion Lowers Intraocular Pressure in Normotensive Dutch-Belted Rabbits

**DOI:** 10.3390/pharmaceutics14122585

**Published:** 2022-11-24

**Authors:** Samir Senapati, Ahmed Adel Ali Youssef, Corinne Sweeney, Chuntian Cai, Narendar Dudhipala, Soumyajit Majumdar

**Affiliations:** 1 Department of Pharmaceutics and Drug Delivery, School of Pharmacy, University of Mississippi, Oxford, MS 38677, USA; 2 Department of Pharmaceutical Technology, Faculty of Pharmacy, Kafrelsheikh University, Kafrelsheikh 33516, Egypt; 3 Research Institute of Pharmaceutical Sciences, University of Mississippi, Oxford, MS 38677, USA

**Keywords:** cannabidiol, nanoemulsion, carbopol^®^ 940 NF, autoclave, IOP, rabbits

## Abstract

Cannabidiol (CBD) is the major non-psychoactive and most widely studied of the cannabinoid constituents and has great therapeutic potential in a variety of diseases. However, contradictory reports in the literature with respect to CBD’s effect on intraocular pressure (IOP) have raised concerns and halted research exploring its use in ocular therapeutics. Therefore, the current investigation aimed to further evaluate CBD’s impact on the IOP in the rabbit model. CBD nanoemulsions, containing Carbopol^®^ 940 NF as a mucoadhesive agent (CBD-NEC), were prepared using hot-homogenization followed by probe sonication. The stability of the formulations post-moist-heat sterilization, in terms of physical and chemical characteristics, was studied for three different storage conditions. The effect of the formulation on the intraocular pressure (IOP) profile in normotensive Dutch Belted male rabbits was then examined. The lead CBD-NEC formulation (1% *w*/*v* CBD) exhibited a globule size of 259 ± 2.0 nm, 0.27 ± 0.01 PDI, and 23.2 ± 0.4 cP viscosity, and was physically and chemically stable for one month (last time point tested) at 4 °C, 25 °C, and 40 °C. CBD-NEC significantly lowered the IOP in the treated eyes for up to 360 min, with a peak drop in IOP of 4.5 mmHg observed at the 150 min time point, post-topical application. The IOP of the contralateral eye (untreated) was also observed to be lowered significantly, but the effect lasted up to the 180 min time point only. Overall, topically administered CBD, formulated in a mucoadhesive nanoemulsion formulation, reduced the IOP in the animal model studied. The results support further exploration of CBD as a therapeutic option for various inflammation-based ocular diseases.

## 1. Introduction

Cannabis contains more than 100 cannabinoids [1]. Δ^9^-tetrahydrocannabinol (THC) is the major psychoactive component while cannabidiol (CBD) is the major non-psychoactive and most widely studied of the other cannabinoid constituents. CBD is known to act on various receptor targets such as peroxisome proliferator-activated receptor gamma (PPARγ) and 5-hydroxytryptamine 1A receptor (5-HT1A), and shows anti-inflammatory (by interacting with the CB_2_ receptor and inhibiting immune cell migration), neuroprotective, and antioxidant properties (by scavenging reactive oxygen species (ROS) and blocking NADPH oxidase) [2,3,4]. CBD, thus, holds tremendous potential as a therapeutic candidate in multiple ocular diseases. 

In recent years, however, contradictory reports in the literature and the lack of rigor in most prior studies make it difficult to conclude the impact of CBD on intraocular pressure (IOP). Concerns about CBD increasing IOP on topical application have generated questions about the safety of CBD for ocular use. A review of the literature reveals that, prior to 2022, a total of nine studies investigated the effect of CBD on the IOP. These investigations were conducted in rabbits, cats, monkeys, and humans, and were administered via topical ocular, oral, sublingual, or intravenous routes of administration. Intravenous CBD administration in rabbits [5,6,7] and oral administration in monkeys [6] did not demonstrate any effect on the IOP. On the other hand, topical application of CBD in rabbits, using mineral-oil-based formulations [8], continuous topical application in cats using a mini pump [9], and intravenous CBD application in human subjects [10] all led to transient drop in IOP. 

Two studies, however, have reported an increase in IOP after CBD application [11,12]. Tomida et al. investigated sublingual administration of CBD in humans through oromucosal spray and observed a transient rise in the IOP with a CBD dose of 40 mg but not with 20 mg [11]. In 2018, a report by Miller et al. raised serious concerns about the safety of topical ocular CBD application [12]. The authors studied the effect of THC (5 mM; 0.16% *w*/*v*) and CBD (5 mM; 0.16% *w*/*v*), alone and in combination, on the IOP in C57BL/6J (C57) mice and CB1 knockout (CB1 KO) mice on CD1 strain background. THC lowered the IOP in C57 male mice for 8 h whereas CBD increased the IOP in C57 male mice. However, when CBD was administered to CB1 KO mice, this resulted in a decrease in IOP 1 h after administration, but there was no effect observed 4 h post-administration. When CBD/THC mixture was administration to C57 mice, CBD blocked the effect of THC and there was no effect on IOP observed in the treated and contralateral eyes (no statistically significant difference). Based on the above observations, Miller et al. concluded that, unlike THC, CBD increases the IOP upon topical application and on co-administration with THC, CBD counteracts the IOP lowering effect of THC. 

Contrary to the observation by Miller et. al. [12], Rebibo et al., in 2022, reported that a CBD nanoemulsion (NE) decreased IOP when administered three times a day for two weeks. At 0.4% CBD concentration, IOP decreased significantly in female C57BL/6 mice after 7 and 14 days compared to the baseline values within the group [13]. A reduction in IOP was also observed with 1.6% CBD after 3 and 14 days of topical application. Additionally, 0.4% CBD significantly lowered the IOP at 7, 10, and 14 treatment days compared to the blank NE group, whereas the 1.6% NE reduced the IOP after 3, 10, and 14 days compared to the blank NE group. However, a reduction in IOP was not seen in the 0.8% CBD treated groups. Thus, although there are some questions in this report as to why the middle dose did not show any effect on the IOP, the data suggests that topically administered CBD did not increase the IOP in mice. 

Thus, except for the two studies conducted by Miller et al. and Tomida et al. [11,12], all other studies indicate that CBD does not increase IOP but rather has an IOP lowering effect. However, based on the report by Miller et al. [12], the development of CBD as a therapeutic candidate for ocular diseases has almost come to a halt because of IOP related safety concerns. 

CBD’s site of action is at the trabecular meshwork or the iris-ciliary (IC) bodies, or both, depending upon the molecular mechanisms involved based on the current understanding [14,15]. Thus, for CBD to mediate local activity following topical application, it has to penetrate across the corneal epithelium and reach the anterior segment ocular tissues, especially the aqueous humor (AH) and IC bodies to achieve a therapeutic effect. Our earlier investigations explored the penetration of CBD into the ocular tissues through the topical route [16]. In that investigation, Tocrisolve-based CBD formulations (0.47% *w*/*v*) were prepared and tested in New Zealand White (NZW) rabbits and the study revealed that the CBD levels in the ocular tissues, 90 min post-administration, were very low [16]. Unfortunately, in that study, the effect of the Tocrisolve--based CBD formulations on the IOP was not monitored. However, to better understand the effect of CBD on the IOP in rabbits, a formulation allowing better delivery of CBD into the intraocular tissues would be needed. 

Previously, we encountered similar transcorneal delivery challenges with THC from mineral oil or Tocrisolve-based THC NE formulations and resultant lack of an IOP lowering effect through the topical administration route [17,18,19]. This ultimately led to the development of a Carbopol^®^ 940 NF containing THC NE formulation (THC-NEC) with low oil (5% instead of the 20% in Tocrisolve) and different surfactant concentrations [20]. With this THC-NEC formulation we were able to demonstrate consistent IOP lowering activity of THC in New Zealand White (NZW) rabbits [20,21]. 

Thus, the first aim of this study was to formulate CBD into the THC-NEC formulation vehicle, to enhance drug retention on the ocular surface and allow better permeation through the corneal membrane [20]. The second aim was to understand the effect of CBD on the IOP of pigmented Dutch Belted (DB) rabbits. The DB rabbits show a higher IOP than the NZW rabbits and thus serve as a better model to study the effect on IOP. Additionally, the role of pigmentation, if any, on the duration of activity would be evident in this model.

## 2. Materials and Methods

### 2.1. Materials

#### 2.1.1. Chemicals and Glassware

Cannabidiol (CBD) derived from hemp was used in this study and was a gift from ElSohly Laboratories, Inc. (Oxford, MS, USA). Optical rotation, melting point test, mass spectroscopy, and nuclear magnetic resonance spectroscopy studies were performed to ensure that this investigation was carried out with the (-) normal CBD isomer. Poloxamer 407 NF grade, sesame oil NF grade, Carbopol^®^ 940 NF grade, glycerin NF grade, and Tween^®^ 80 (Polysorbate 80) NF grade were purchased from Spectrum Chemicals (New Brunswick, NJ, USA). All other chemicals, including Vitamin E-d-alpha-tocopherol polyethylene glycol 1000 succinate (TPGS), were purchased from Fisher Scientific (St. Louis, MO, USA). Solvents used for the instrumental analysis were of high-performance liquid chromatography (HPLC) grade and were purchased from Fischer Scientific (St. Louis, MO, USA). Centrifuge tubes, scintillation glass vials, and Slide-A-Lyzer™ MINI Dialysis Devices (10 K molecular weight cutoff) were purchased from Fischer Scientific (Hampton, NH, USA). Screw top clear class A (Type I) borosilicate glass HPLC vials (12 × 32 mm, 2.0 mL) with pre-slit silicone septa were purchased from Waters (Waters, Milford, CA, USA). 

#### 2.1.2. Animals

Dutch Belted (DB) male rabbits (weight; 4.75–5.75 lbs and age; 8–12 weeks) were purchased from ENVIGO (Denver, PA, USA). All animal experiments conformed to the tenets of the Association for Research in Vision and Ophthalmology statement on the Use of Animals in Ophthalmic and Vision Research and followed the University of Mississippi Institutional Animal Care and Use Committee approved protocols (18-029). 

### 2.2. Methods

#### 2.2.1. HPLC Method

CBD was quantified using an HPLC-UV system comprising of a Waters^®^ Alliance e2695 separations module and a Waters^®^ 2489 UV/Vis dual absorbance detector. Stock solutions of CBD were prepared in acetonitrile. A detection wavelength (λ_max_) of 210 nm was set. The mobile phase, consisting of a mixture of solution A and B (75:25 *v*/*v*), was pumped isocratically at a flow rate of 1.2 mL/min. Solution A consisted of a mixture of acetonitrile and methanol (75:25 *v*/*v*) containing 0.05% *v*/*v* formic acid, while solution B was Milli-Q water containing 0.05% *v*/*v* trifluoroacetic acid. Chromatographic separation was achieved within 10 min on a Waters Symmetry^®^ C18 column (150 × 4.6 mm, 5 μm) as a stationary phase with a retention time of 7.5 min. The injection volume was set to 20 μL and the detector sensitivity was set to 2.0 AUFS (absorbance units full scale). The column temperature was kept at 40 °C while the sample holder temperature was kept at 25 °C. The HPLC method was linear over a CBD concentration range of 1–20 μg/mL.

#### 2.2.2. Preparation of CBD-NE Formulations

The composition of the CBD-NE formulation is presented in Table 1. Oil in water (O/W) NE was prepared using hot homogenization followed by the probe sonication method [22,23]. The oil phase was prepared by dissolving an accurately weighed amount of CBD in sesame oil, with heating at 70 ± 2 °C, to obtain a clear oily solution. The aqueous phase comprising glycerin (tonicity adjusting agent), Poloxamer 407 and Tween^®^ 80 (surfactants), TPGS (antioxidant), and water was placed in another glass vial and simultaneously heated at 70 °C under continuous stirring in a water bath. The hot aqueous phase was then added to the heated oil phase dropwise under continuous magnetic stirring at 2000 rpm for 5 min to form pre-emulsion. This pre-emulsion was then homogenized using a T25 digital Ultra-Turrax (IKA Works, Inc., Wilmington, NC, USA) at 11,000 rpm for 5 min at 65.0 ± 2.0 °C to form a coarse emulsion. The coarse emulsion was allowed to cool at room temperature before being subjected to probe sonication in an ice bath for 10 min with a 3 mm stepped microtip at 500 watts power supply and 115 volts (40% amplitude, pulse on: 10 s and pulse off: 15 s) using a Sonics Vibra-Cell™ Sonicator (Newtown, CT, USA) to form NE. 

#### 2.2.3. Preparation of the Mucoadhesive CBD-NE (CBD-NEC)

The NE preparation method, as described above, was followed for the preparation of the NEC formulations (Table 1). However, the volume of Milli-Q water used to prepare NE was split into two equal parts: one part was used to prepare the aqueous solution of the mucoadhesive agent (Carbopol^®^ 940 NF) and the other half was used to prepare the aqueous phase as described above. They were both heated to 70 °C and added into the oily phase containing the drug in a drop-by-drop fashion, using two different pipettes, under constant magnetic stirring at 2000 rpm for 5 min to form a pre-emulsion. This pre-emulsion was then homogenized using a T25 digital Ultra-Turrax (IKA Works, Inc., Wilmington, NC, USA) at 11,000 rpm for 5 min at 65.0 ± 2.0 °C to form a coarse emulsion. The coarse emulsion was allowed to cool at room temperature before being subjected to probe sonication in an ice bath for 10 min with a 3 mm stepped microtip at 500 watts power supply and 115 volts (40% amplitude, pulse on: 10 s and pulse off: 15 s) using Sonics Vibra-Cell™ Sonicator (Newtown, CT, USA) to form NE [21,24]. 

#### 2.2.4. Measurement of Globule Size, Polydispersity Index (PDI), and Zeta Potential (ZP)

The NE and NEC formulations were evaluated for their globule size, PDI, and ZP using Zetasizer (Nano ZS Zen3600, Malvern Panalytical Inc., Westborough, MA, USA) at 25 °C in disposable, folded, clear, and solvent-resistant micro-cuvettes (ZEN0040). The NE and NEC formulations were diluted (100-fold) with Milli-Q water [25]. The same diluted formulations were transferred to Zetasizer disposable folded capillary DTS1070 cells for performing zeta potential measurement after globule size and PDI measurement [26,27]. All globule size, PDI, and zeta potential measurements were conducted in triplicate. 

#### 2.2.5. Assay (CBD Content)

Fifty microliters (50 μL) of the CBD-NE and CBD-NEC formulations were transferred into a volumetric flask (5 mL) and the volume was adjusted with acetonitrile (extracting solvent, 100-fold dilution). The extract was vortexed (5 min, 2000 rpm, Vortex-Genie^®^ 2, Scientific Industries, Inc., Bohemia, NY, USA) and sonicated (Bransonic^®^ ultrasonic cleaner, Branson Ultrasonics corporation, Brookfield, CT, USA) for 10 min. The extract was then centrifuged (AccuSpin 17R centrifuge, Fisher Scientific, Hanover, IL, USA) for 20 min at 13,000 rpm. Then, the supernatant was diluted (10-fold) with acetonitrile before being analyzed for CBD content using the HPLC method mentioned above. 

#### 2.2.6. pH Measurement

The pH was measured with a Mettler Toledo pH meter (FiveEasy™, Columbus, OH, USA) equipped with an Inlab^®^ Micro Pro-ISM probe. Before measurement, the pH meter was calibrated using different buffers with known pH values of 4.01, 7.00, and 10.01 (Orion™ Standard All-in-One™ pH Buffer Kit, Thermo Fisher Scientific, Chelmsford, MA, USA). The pH measurements were carried out in triplicate.

#### 2.2.7. Viscosity Measurement

A Brookfield cone and plate viscometer (LV-DV-II+ Pro Viscometer, Middleborough, MA, USA) was used to measure the viscosity of the prepared NE and NEC formulations in the presence and absence of simulated tear fluid (STF). STF was prepared by dissolving 0.0084% calcium chloride, 0.138% potassium chloride, 0.678% sodium chloride, and 0.218% sodium bicarbonate in Milli-Q water and the pH was adjusted to 7.0 ± 0.2 with 0.1 N hydrochloric acid. STF was mixed with the formulation in a ratio of 7:50 prior to viscosity measurement. The STF to formulation ratio was selected based on the expectation that a standard eyedropper dispenses 0.05 mL (50 µL) while the precorneal tear fluid volume is about 7 to 10 μL [25]. The cup was thermally equilibrated at 25 ± 0.5 °C for 15 min with the aid of a circulating water bath before the test. The sample (0.5 mL) was placed in the viscometer cup plate and rotated at a constant speed of 5.0 rpm using a CPE 52 spindle. The torque required to maintain this speed was measured and translated to viscosity values (centipoise, cP). Rheology analysis was performed using Rheocalc^®^ software (Version 3.3 Build 49-1, USA). The viscosity measurements of all samples were carried out in triplicate.

#### 2.2.8. Sterilization Process and Stability Assessment

CBD-NE and CBD-NEC formulations were subjected to terminal moist heat sterilization (121 °C under 15 psi for 15 min, 3850ELP-B/L-D Tuttnauer autoclave, Heidolph, Germany) process in glass vials. The sterilization cycle was confirmed by the color change of the indicator tapes attached to the glass vials. 

#### 2.2.9. Stability Studies

The physicochemical stability of NE and NEC formulations was evaluated at refrigerated (4 ± 2 °C), room temperature (25 ± 2 °C), and accelerated (40 °C ± 2 °C) storage conditions pre- and post-moist heat sterilization process. The formulations were evaluated for any change in globule size, PDI, ZP, pH, and CBD content over the one month of storage. 

#### 2.2.10. Scanning Transmission Electron Microscopy (STEM) 

The analysis was carried out using a JSM-7200FLV Scanning Electron Microscope (JOEL, Peabody, MA, USA) attached to a STEM detector with an accelerating voltage of 30 kV. STEM samples were examined following a negative staining protocol with a solution of UranyLess. A carbon-plated copper grid was placed on top of one drop (20 µL) of the formulation for 60 s and the excess sample was blotted off with a filter paper after grid removal from the formulation surface to remove any excess sample. The grid was then washed by dipping it in distilled water and the grid was blotted with filter paper to remove excess water. Next, the grid was placed sample down on top of one drop (20 µL) of the staining solution for 60 s and then was blotted with a filter paper to remove any excess stain. The grid was allowed to dry for a few minutes by air. The grid was examined under the scanning transmission electron microscope at 30 K times magnification power.

#### 2.2.11. In Vivo Single Dose Efficacy Studies—IOP Measurement

IOP was measured following our earlier published protocols [20,21]. Pigmented DB rabbits were acclimatized to the environment, personnel, and measurement procedure for 14 days to establish the IOP baseline. The CBD-NEC and NEC placebo formulations were individually instilled (50 µL) into the lower cul-de-sac of the left eye (treated eye) of the DB rabbits, while the right eye (contralateral) was kept untreated. Following topical instillation, the eyelids were kept closed (5–10 s) to decrease spillage of the NE formulations. The IOP was measured before instillation (to establish a baseline IOP) and every 30 min up to 180 min, and then every 60 min up to 480 min post-instillation. The IOP value displayed on the TONOVET Plus tonometer (Icare^®^ Finland Oy, Finland) was an average of six readings and at each time point was measured in triplicate. The IOP measurements at each time point are reported as percent change in IOP (Equation (1)).

(1) $$\Delta \mathrm{IOP}=\frac{\;\mathrm{Baseline}\;\mathrm{IOP}\;-\;\mathrm{Measured}\;\mathrm{IOP}}{\mathrm{Baseline}\;\mathrm{IOP}}\times 100$$

Ocular irritation was qualitatively evaluated through visual monitoring of redness and changes in tearing or blinking rate. 

#### 2.2.12. Statistical Analysis

Statistical comparisons of the means were performed using one-way analysis of variance (ANOVA). The difference was considered significant when the *p*-value was <0.05. SPSS 28 software (IBM^®^, Armonk, NY, USA) was used for the statistical evaluations. 

## 3. Results and Discussion

NE formulations are nanosized (20–200 nm), thermodynamically stable, isotropic systems [22]. NEs have a high solubilizing capacity for therapeutic moieties, excellent physicochemical stability, and are biocompatible [28,29]. NEs can improve ocular permeation, extend drug release, and enable drug distribution to the deeper ocular tissues [22,28]. The low surface tension of these nanocarriers facilitate excellent spreading on the ocular surface and excellent mixing with the tear film, thus prolonging the residence time of the drug on the ocular surface and improving ocular permeation [30]. All these factors led to considering these nanocarriers as the vehicle of choice for this investigation.

Mucoadhesive agents prolong the ocular surface residence time of topically applied therapeutics by adhering to the mucin layer of the tear film, thus enabling uniform distribution of the applied eyedrops above the cornea [20,31]. Therefore, the addition of a mucoadhesive agent to the developed NE could result in prolonging the activity and improving the therapeutic outcomes of CBD. Many mucoadhesive agents that have been reported and used in many Food and Drug Administration (FDA) approved ophthalmic dosage forms such as chitosan, Carbopol^®^, hydroxyl propyl methyl cellulose, sodium carboxy methyl cellulose, hyaluronan, and xanthan gum. 

Carbomer copolymer type A (Pemulen TR-2 NF Carbopol^®^ 71G NF, Carbopol^®^ 971P NF, and Carbopol^®^ 981 NF) and B (Pemulen TR-1 NF) and Carbomer homopolymer type B (Carbopol^®^ 974P NF, Carbopol^®^ 5984 EP, and Carbopol^®^ 934 NF) and C (Carbopol^®^ 940 NF, and Carbopol^®^ 980 NF) have been used in many FDA approved ophthalmic formulations [20]. Carbomer homopolymer type C (Carbopol^®^ 940 NF) gives the highest viscosity at pH 7.5 and has been used up to 4.0% *w*/*w* in FDA approved ophthalmic formulations according to the FDA inactive ingredients database. Besides its mucoadhesive properties, Carbopol^®^ 940 NF undergoes a pH-dependent *sol-to-gel* transition to form a viscoelastic gel above a pH of 5.5. The normal pH range of human tear fluid is 6.5 to 7.6 with an average value of 7.0 [23,32]. In the alkaline environment of human tears, the carboxyl groups ionize and generate many negative charges along the Carbopol^®^ polymer backbone. The electrostatic repulsion of the similar negative charges of the anionic carboxyl group triggers the uncoiling and expansion of the polymeric chains, thus resulting in polymer swelling and gel formation [33].

Our earlier investigation studied the effect of inclusion of Carbopol^®^ 940 NF on the intensity and duration of IOP lowering activity of Δ^9^ -tetrahydrocannabinol-valine-hemisuccinate (THC-VHS) loaded in a nanoemulsion formulation [20]. The THC-VHS-NEC formulation demonstrated a significant (*p* < 0.05) improvement in the duration of IOP lowering activity, compared to THC-VHS-NE. Moreover, THC-VHS-NEC was more effective than the marketed latanoprost ophthalmic eyedrops in terms of both duration and intensity of IOP lowering. Thus, with the main objective of increased ocular surface residence of the topically applied eye drops, Carbopol^®^ 940 NF was the best choice for this study with its dual mechanism for serving the objective. 

### 3.1. Formulation Development

CBD-NE and CBD-NE formulations were prepared according to our previously established protocols [20]. The composition of the prepared NE formulations is presented in Table 1. The NE formulations were prepared using sesame oil as the oily phase as CBD possesses adequate solubility in this oil. Moreover, Epidiolex^®^, the only FDA approved commercial product (oral) for CBD, is provided as a solution in sesame oil. The selected Carbopol^®^ 940 NF concentration (0.4% *w*/*w*) increases the viscosity of the prepared NE while facilitating easy topical ocular application (≤50 cP) based on our earlier investigations [20].

### 3.2. Physicochemical Characteristics of CBD-NE and CBD-NEC Formulations

The globule size, PDI, ZP, and drug content of CBD-NE and CBD-NEC formulations are illustrated in Table 2. The globule size is an important factor for adhesion and interaction with ocular epithelial cells. The reduction in globule size provides a larger surface area for contact with the ocular surface, which can significantly enhance bioavailability [34]. Smaller globules (<200 nm) are transported by receptor-mediated endocytosis uptake whereas larger particles are transported by phagocytosis [23]. PDI values show the width of globule size distribution and PDI values < 0.3 demonstrate that the NE formulations were uniform dispersions with narrow globule size distribution [35]. ZP values of more than ±30 mV indicate good physical stability for the prepared NEs [23,36]. 

Carbopol^®^ 940 NF addition changed ZP significantly from −19.8 ± 1.1 for CBD-NE to −37.9 ± 0.8 for CBD-NEC, probably due to the negatively charged carboxylic acid groups along the polymer backbone. The change in ZP suggests that Carbopol^®^ 940 NF is adsorbed on the surface of the oil droplets [20]. The significant increase (*p* < 0.05) in globule size from 167.2 ± 4.2 for CBD-NE to 259.5 ± 2.0 for CBD-NEC also supports surface adsorption of Carbopol^®^ 940 NF [20]. Both NE formulations demonstrated CBD content, 100.0 ± 0.3 for CBD-NE and 101.9 ± 0.1 for CBD-NEC, within the acceptance limits of the label’s content (90–110%).

### 3.3. pH and Viscosity

Although physiological pH ranges are always preferred, the eye can tolerate topical formulations with low buffering capacity over a pH range of 3.0–8.6. In this study, incorporation of the mucoadhesive agent, Carbopol^®^ 940 NF, significantly (*p* < 0.05) affected the pH of the formulation (Table 2). The decrease in pH from 5.6 ± 0.02 (CBD-NE) to 3.6 ± 0.02 (CBD-NEC) would be due to the acidic carboxylic groups of Carbopol^®^ 940 NF. The pH values of both formulations were within the acceptable range. Moreover, in our earlier studies with THC-VHS using the same vehicle, there were no signs of discomfort, ocular irritation, or redness in the treated eye during the in vivo single-dose efficacy study period (8 h study) based on visual examination [20]. This suggests the lower pH of the CBD-NEC formulation would not cause any ocular irritation or redness in the treated eye. 

Viscosity is a critical parameter of topical ophthalmic dosage forms because it can affect the performance of the applied product as well as patient comfort. Generally, viscosity values up to 50 cP have been established to be most favorable in terms of patient compliance, ease of topical ocular application, and prolonging retention at the ocular surface, thus leading to improved ocular bioavailability [37]. The viscosity of both NE formulations was measured using a Brookfield cone and plate viscometer (Table 2). It was observed that the viscosity of the CBD-NE formulation (11.6 ± 0.5 cP) was significantly (*p* < 0.05) increased, expectedly, after the incorporation of Carbopol^®^ 940 NF (CBD-NEC; 23.2 ± 0.4 cP) within the formulation. Moreover, the addition of STF increased the viscosity (31.2 ± 1.2) of CBD-NEC significantly (*p* < 0.05) because the alkaline environment (7.0 ± 0.2) resulted in Carbopol^®^ 940 NF swelling and gel formation. All measured viscosity values were favorable for ocular application.

### 3.4. STEM

**Figure 1:**
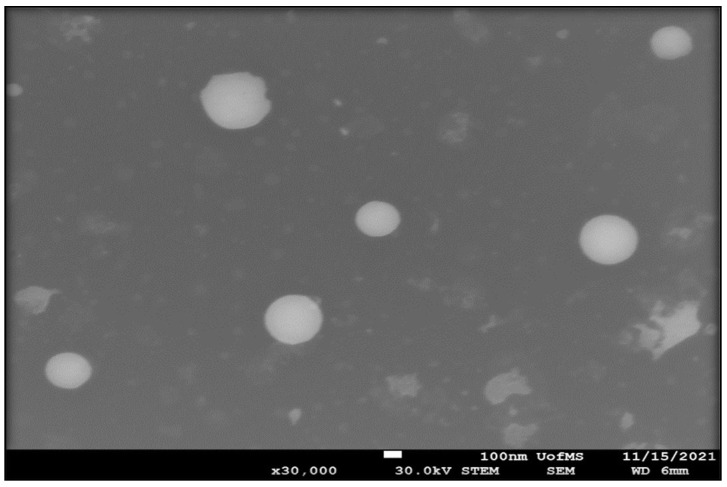
STEM micrograph of CBD-NEC formulation.

The surface morphology of CBD-NEC was studied using STEM and the result is shown in Figure 1. The globules were spherical in shape with a globule size around 250 d.nm, which is consistent with the results obtained from dynamic light scattering studies. 

### 3.5. Stability Studies

The physicochemical stability of both NE formulations was evaluated at 4, 25, and 40 °C storage conditions over 30 days (last time point tested). Both formulations did not show any change in color, precipitation, creaming, or cracking during the testing period upon visual examination. 

Addition of Carbopol^®^ 940 NF makes filtration of the NEC formulation very challenging, necessitating aseptic manufacturing if not autoclavable. Terminal sterilization of the final dosage form is always more preferred than aseptic manufacturing. Thus, moist-heat sterilization was also investigated for the NE and NEC formulations. The pre- and post-moist-heat sterilization physicochemical characteristics are provided in Table 3. The autoclaved formulations remained stable under the test conditions employed; globule size, PDI, ZP, pH, and drug content did not show a significant (*p* < 0.05) change in comparison to the pre-autoclaved formulation for one month.

Surfactant composition, globule size, and surface charge are directly related to the physical stability of NEs. The combined effect of these three parameters determines the formulation stability. A suitable surfactant combination provides an elastic interface between the two immiscible liquids, allowing the dispersed phase to be suspended in the form of small globules in the dispersion medium [38]. The oil globules become elastic and can survive a high degree of tension during deformation. In addition, smaller dispersed oil globules are not affected by gravitational force and become suspended continuously in the dispersion medium [39]. Moreover, numerous surface charges over the smaller oil globules keep them separate due to the presence of strong interglobular repulsive forces [40]. 

The continuous motion of oil globules within the dispersion medium makes oil globules approach each other, and the globules become subjected to strong repulsive force and finally move apart after the elastic collision [40]. This phenomenon improves kinetic stability by Brownian motion [38]. Although high-molecular-weight surfactants such as Tween^®^ 80 and Poloxamer 407 provide a lower magnitude of ZP, these surfactants keep the NE stable by the additive effects of steric hindrance and the repulsive force between similarly charged globules [38]. Based on the HLB theory reported by Griffin, a mixture of surfactants with a final HLB value between 9 and 12 is sufficient to prepare stable O/W Nes [41,42]. Hence, it seems that the 1:10 ratio of Poloxamer 407: Tween^®^ 80 (HLB: ~15.6) could provide excellent physical stability. It is worth mentioning that the same formulation composition loaded with THC-VHS provided excellent stability with respect to globule size, PDI, ZP, pH, and drug content pre- and post-sterilization in our earlier investigation [20].

### 3.6. In Vivo Single-Dose Efficacy Studies—IOP Measurement

In earlier studies, Miller et al. reported there was an increase in IOP in mice after administering CBD [12], whereas Rebibo et al. [13] reported a decrease in IOP when mice were dosed with a CBD emulsion. Both groups administered a blank vehicle to the contralateral eye and a drug loaded formulation to the treated eye; however, they report contrasting results. This could be attributed to how the change in IOP was determined. Miller et al. compared the IOP of the treated eye to that of the contralateral eye, whereas Rebibo et al. [13] compared the IOP of the treated eye to the baseline values. As the literature indicates, IOP lowering agents applied to one eye can significantly reduce IOP in the contralateral eye also [17,20,43,44,45,46,47,48]. This has also been observed in the mouse model [49,50]. CBD, like THC, is seen to lower the IOP in the contralateral eye. Thus, concluding the effect of CBD on the IOP, or a change in IOP, based on a comparison between the contralateral and treated eye IOPs could be erroneous. 

Miller et al. and Rebibo et al. both used a mouse model to demonstrate the impact of CBD on IOP. The mouse model is particularly attractive due to the ease of husbandry, the extensive genomic resources available, and the potential for genetic manipulation [51]. However, the mouse model for glaucoma also presents challenges with obtaining an accurate determination of IOP due to the small size of the mouse eye. Both research groups have used anesthesia to measure the IOP. Anesthesia has been reported to influence the IOP in rodents. The type and quantity of the anesthesia used determines how much the IOP is influenced [52]. Kim et al. reported that the mean IOP decreases rapidly during the first 10 min after the loss of the lid reflex and remains unchanged thereafter. Both groups have reported measurements taken after the successful induction of anesthesia, which could impact the IOP reading [12,13]. 

**Figure 2:**
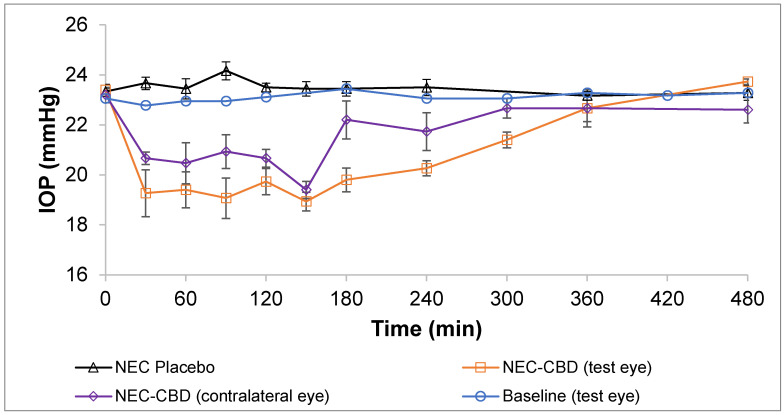
IOP vs. time profile following topical administration of CBD-NEC (treated eye and contralateral eye), NEC placebo (treated eye), and baseline of left eye in Dutch Belted male rabbits (mean ± SEM, *n* = 6).

Figure 2 illustrates the IOP vs. time profile following topical administration of CBD-NEC (treated eye and contralateral eye), NEC placebo (treated eye), and baseline of left eye in Dutch Belted male rabbits. The IOP versus time profile of the baseline of the left eye (test eye) was established prior to application of NEC formulations. There were no significant (*p* > 0.05) differences found in the baseline of the test eye over the 8 h and an average IOP of 23.1 ± 0.2 mmHg was maintained. The NEC-placebo formulation exhibited a similar IOP vs. time profile as the baseline and maintained an average IOP of 23.5 ± 0.3 mmHg. The CBD-NEC formulation demonstrated a significant IOP-lowering effect in the DB rabbits following a topical application (*p* < 0.05). A drop of more than 10% was observed for both the contralateral and test eye 30 min after the application of CBD-NEC formulation in the test eye as well as the contralateral eye. The maximum drops in IOP (% drop from time 0) for the test eye and contralateral eye after the instillation of CBD-NEC were 19.9% and 17.4%, respectively, at 150 min post-application. The durations of action (considered only if the drop in IOP was more than 10% from baseline) for the test eye and contralateral eye were 240 min and 150 min, respectively. The IOP began returning to baseline after 180 min post-application. The IOP of the treated eye after application of CBD-NEC was significantly lower than the established left eye baseline from the 30 min time point (*p* < 0.005) and this effect lasted until 300 min (*p* < 0.001). The statistical difference between the treatments can be found in Table 4. Based on previous data that suggest the addition of the mucoadhesive agent to a nanoemulsion prolongs the duration of action compared to the nanoemulsion alone, CBD-NE was not tested [20]. 

## 4. Conclusions

This study is the first to report—to the author’s knowledge—that CBD lowers the IOP in normotensive Dutch Belted rabbits following topical application. The CBD-NEC formulation exhibited a max drop of 19.9% with a duration of 300 min. The CBD-NE and CBD-NEC formulations were successfully prepared using Carbopol^®^ 940 NF as a mucoadhesive agent and were evaluated for stability at 4, 25, and 40 °C. Both formulations were sterilized via moist heat and the pre- and post-autoclaved formulations were stable for at least one month (last time point tested) after the sterilization process. The ocular tissue biodistribution profile, dose–effect relationship, effect of sex on the pharmacodynamic activity, and investigations into the IOP lowering mechanism are the topics of additional studies that are underway. In summary, the prepared NE formulations could provide a promising CBD delivery platform for the treatment of glaucoma or other ocular indications.

## Figures and Tables

**Table 1:** Composition of cannabidiol-loaded nanoemulsion formulations.

Ingredients (% *w*/*v*)	Formulations	
	CBD-NE	CBD-NEC
CBD	1.0	1.0
Sesame oil NF	5.0	5.0
Tween^®^ 80 NF	2.0	2.0
Poloxamer 407 NF	0.2	0.2
Glycerin NF	2.25	2.25
Carbopol^®^ 940 NF	–	0.4
TPGS	0.002	0.002
Milli-Q water q.s (mL)	10	10

**Table 2:** Globule size, PDI, zeta potential, drug content, pH, and viscosity of the CBD-NE and CBD-NEC formulations (mean ± SD, *n* = 3).

Parameter	Formulation	
	CBD-NE	CBD-NEC
Globule size (d. nm)	167.2 ± 4.2	259.5 ± 2.0
Polydispersity index	0.20 ± 0.01	0.27 ± 0.01
Zeta potential (mV)	−19.8 ± 1.1	−37.9 ± 0.8
Drug content (%)	100.0 ± 0.3	101.9 ± 0.1
pH	5.6 ± 0.02	3.6 ± 0.02
Viscosity (cP) without STF	11.6 ± 0.5	23.2 ± 0.4
Viscosity (cP) with STF	12.3 ± 1.0	31.2 ± 1.2

**Table 3:** Stability data for CBD-NE and CBD-NEC nanoemulsion formulations pre- and post- sterilization over one month of storage at 4, 25, and 40 °C (mean ± SD, *n* = 3).

Formulation	Day	Storage at 4 ± 2 °C									
		DS (d, nm)		PDI		ZP (mV)		pH		Drug Content (%)	
		Sterilization Stage									
		Pre	Post	Pre	Post	Pre	Post	Pre	Post	Pre	Post
CBD-NE	0	167.2 ± 4.2	167.9 ± 2.0	0.20 ± 0.01	0.21 ± 0.02	−19.8 ± 1.1	−21.6 ± 0.2	5.60 ± 0.02	5.62 ± 0.01	100.0 ± 0.3	97.0 ± 0.2
	30	166.0 ± 3.7	163.6 ± 2.6	0.20 ± 0.02	0.23 ± 0.01	−21.7 ± 0.5	−20.6 ± 0.4	5.60 ± 0.02	5.64 ± 0.02	99.5 ± 2.1	99.0 ± 0.5
CBD-NEC	0	259.5 ± 2.0	258.5 ± 0.4	0.26 ± 0.01	0.25 ± 0.02	−33.9 ± 0.8	−34.7 ± 0.3	3.60 ± 0.01	3.59 ± 0.03	101.9 ± 0.1	98.2 ± 0.3
	30	252.9 ± 2.8	251.7 ± 3.7	0.25 ± 0.01	0.26 ± 0.01	−35.2 ± 0.2	−33.6 ± 0.4	3.60 ± 0.01	3.65 ± 0.01	101.9 ± 1.4	102.6 ± 1.5
		Storage at 25 ± 2 °C									
CBD-NE	0	165.7 ± 1.8	166.4 ± 2.0	0.21 ± 0.01	0.22 ± 0.01	−19.8 ± 0.9	−21.6 ± 0.8	5.62 ± 0.02	5.63 ± 0.01	99.8 ± 0.1	97.0 ± 0.1
	30	164.9 ± 1.0	161.9 ± 3.0	0.22 ± 0.01	0.22 ± 0.02	−21.2 ± 0.3	−20 ± 0.6	5.64 ± 0.02	5.62 ± 0.01	98.2 ± 1.7	97.6 ± 0.6
CBD-NEC	0	262.9 ± 1.3	259.4 ± 0.8	0.25 ± 0.02	0.24 ± 0.02	−34.3 ± 2.1	−35.7 ± 0.8	3.61 ± 0.01	3.60 ± 0.02	100.8 ± 0.1	101.0 ± 0.1
	30	256.8 ± 4.7	256.1 ± 0.8	0.26 ± 0.01	0.26 ± 0.01	−35.8 ± 0.6	−34.3 ± 0.8	3.64 ± 0.01	3.65 ± 0.02	100.2 ± 2.0	99.6 ± 2.1
		Storage at 40 ± 2 °C									
CBD-NE	0	166.8 ± 2.2	170.2 ± 2.4	0.19 ± 0.01	0.21 ± 0.01	−21.2 ± 0.5	−19.6 ± 0.3	5.55 ± 0.01	5.64 ± 0.02	99.6 ± 0.1	98.0 ± 0.3
	30	160.9 ± 2.0	162.2 ± 2.6	0.21 ± 0.01	0.21 ± 0.00	−20.8 ± 0.6	−20.6 ± 0.5	5.58 ± 0.01	5.62 ± 0.01	100.3 ± 3.1	99.8 ± 0.2
CBD-NEC	0	263.1 ± 1.8	256.9 ± 2.9	0.24 ± 0.03	0.25 ± 0.01	−34.4 ± 2.1	−35.4 ± 0.3	3.60 ± 0.03	3.62 ± 0.01	99.9 ± 0.2	101.6 ± 0.5
	30	254.9 ± 4.0	254.7 ± 1.8	0.25 ± 0.01	0.26 ± 0.01	−34.6 ± 1.6	−36.2 ± 0.8	3.64 ± 0.01	3.65 ± 0.03	102.9 ± 0.3	102.4 ± 1.2

**Table 4:** *p*-values associated with the difference in the IOP values of treated and contralateral eyes from the baseline value post-instillation of CBD-NEC or placebo formulations in the Dutch Belted male rabbits (*n* = 6).

Time Point (min)	CBD-NEC Treated Eye vs. NEC-Placebo	CBD-NEC Treated Eye vs. CBD-NEC Contralateral Eye	CBD-NEC Treated Eye vs. Baseline Treated Eye	CBD-NEC Contralateral Eye vs. Baseline Treated Eye
	*p*-Value	*p*-Value	*p*-Value	*p*-Value
0	0.211	0.625	1.000	1.000
30	0.003	0.235	0.005	<0.001
60	0.003	0.397	0.001	0.024
90	<0.001	0.155	0.001	0.024
120	<0.001	0.226	<0.001	0.001
150	<0.001	0.431	<0.001	<0.001
180	<0.001	0.045	<0.001	0.309
240	<0.001	0.144	<0.001	0.149
300	–	0.051	<0.001	0.356
360	0.566	1.000	0.271	0.564
480	0.323	0.088	0.045	0.396

## Data Availability

The data presented in this study are available upon request from the corresponding author.

## References

[1] Radwan M.M., ElSohly M.A., Slade D., Ahmed S.A., Khan I.A., Ross S.A. (2009). Biologically Active Cannabinoids from High-Potency Cannabis Sativa. J. Nat. Prod..

[2] De Almeida D.L., Devi L.A. (2020). Diversity of Molecular Targets and Signaling Pathways for CBD. Pharmacol. Res. Perspect..

[3] El-Remessy A.B., Al-Shabrawey M., Khalifa Y., Tsai N.-T., Caldwell R.B., Liou G.I. (2006). Neuroprotective and Blood-Retinal Barrier-Preserving Effects of Cannabidiol in Experimental Diabetes. Am. J. Pathol..

[4] Liou G.I., Auchampach J.A., Hillard C.J., Zhu G., Yousufzai B., Mian S., Khan S., Khalifa Y. (2008). Mediation of Cannabidiol Anti-Inflammation in the Retina by Equilibrative Nucleoside Transporter and A2A Adenosine Receptor. Investig. Ophthalmol. Vis. Sci..

[5] Green K., Symonds C.M., Oliver N.W., Elijah R.D. (1982). Intraocular Pressure Following Systemic Administration of Cannabinoids. Curr. Eye Res..

[6] Elsohly M.A., Harland E.C., Benigni D.A., Waller C.W. (1984). Cannabinoids in Glaucoma II: The Effect of Different Cannabinoids on Intraocular Pressure of the Rabbit. Curr. Eye Res..

[7] Liu J.H., Dacus A.C. (1987). Central Nervous System and Peripheral Mechanisms in Ocular Hypotensive Effect of Cannabinoids. Arch. Ophthalmol..

[8] Green K., Wynn H., Bowman K.A. (1978). A Comparison of Topical Cannabinoids on Intraocular Pressure. Exp. Eye Res..

[9] Colasanti B.K., Powell S.R., Craig C.R. (1984). Intraocular Pressure, Ocular Toxicity and Neurotoxicity after Administration of Δ9-Tetrahydrocannabinol or Cannabichromene. Exp. Eye Res..

[10] Grotenhermen F. (2003). Clinical Pharmacokinetics of Cannabinoids. J. Cannabis Ther..

[11] Tomida I., Azuara-Blanco A., House H., Flint M., Pertwee R.G., Robson P.J. (2006). Effect of Sublingual Application of Cannabinoids on Intraocular Pressure: A Pilot Study. J. Glaucoma.

[12] Miller S., Daily L., Leishman E., Bradshaw H., Straiker A. (2018). Δ9-Tetrahydrocannabinol and Cannabidiol Differentially Regulate Intraocular Pressure. Investig. Ophthalmol. Vis. Sci..

[13] Rebibo L., Frušić-Zlotkin M., Ofri R., Nassar T., Benita S. (2022). The Dose-Dependent Effect of a Stabilized Cannabidiol Nanoemulsion on Ocular Surface Inflammation and Intraocular Pressure. Int. J. Pharm..

[14] Vallée A., Lecarpentier Y., Vallée J.-N. (2021). Cannabidiol and the Canonical WNT/β-Catenin Pathway in Glaucoma. Int. J. Mol. Sci..

[15] Straiker A., Miller S. (2018). Δ9-THC and CBD Differentially Regulate Intraocular Pressure. Investig. Ophthalmol. Vis. Sci..

[16] Taskar P., Adelli G., Patil A., Lakhani P., Ashour E., Gul W., ElSohly M., Majumdar S. (2019). Analog Derivatization of Cannabidiol for Improved Ocular Permeation. J. Ocul. Pharmacol. Ther..

[17] Taskar P.S., Patil A., Lakhani P., Ashour E., Gul W., ElSohly M.A., Murphy B., Majumdar S. (2019). Δ9-Tetrahydrocannabinol Derivative-Loaded Nanoformulation Lowers Intraocular Pressure in Normotensive Rabbits. Trans. Vis. Sci. Tech..

[18] Adelli G.R., Bhagav P., Taskar P., Hingorani T., Pettaway S., Gul W., ElSohly M.A., Repka M.A., Majumdar S. (2017). Development of a Δ9-Tetrahydrocannabinol Amino Acid-Dicarboxylate Prodrug with Improved Ocular Bioavailability. Investig. Ophthalmol. Vis. Sci.

[19] Hingorani T., Adelli G.R., Punyamurthula N., Gul W., ElSohly M.A., Repka M.A., Majumdar S. (2013). Ocular Disposition of the Hemiglutarate Ester Prodrug of ∆9-Tetrahydrocannabinol from Various Ophthalmic Formulations. Pharm. Res..

[20] Sweeney C., Dudhipala N., Thakkar R., Mehraj T., Marathe S., Gul W., ElSohly M.A., Murphy B., Majumdar S. (2022). Impact of Mucoadhesive Agent Inclusion on the Intraocular Pressure Lowering Profile of Δ9-Tetrahydrocannabinol-Valine-Hemisuccinate Loaded Nanoemulsions in New Zealand White Rabbits. Int. J. Pharm..

[21] Sweeney C., Dudhipala N., Thakkar R., Mehraj T., Marathe S., Gul W., ElSohly M.A., Murphy B., Majumdar S. (2021). Effect of Surfactant Concentration and Sterilization Process on Intraocular Pressure–Lowering Activity of Δ9-Tetrahydrocannabinol-Valine-Hemisuccinate (NB1111) Nanoemulsions. Drug Deliv. Transl. Res..

[22] Youssef A.A.A., Cai C., Dudhipala N., Majumdar S. (2021). Design of Topical Ocular Ciprofloxacin Nanoemulsion for the Management of Bacterial Keratitis. Pharmaceuticals.

[23] Youssef A.A.A., Thakkar R., Senapati S., Joshi P.H., Dudhipala N., Majumdar S. (2022). Design of Topical Moxifloxacin Mucoadhesive Nanoemulsion for the Management of Ocular Bacterial Infections. Pharmaceutics.

[24] Tatke A., Dudhipala N., Janga K.Y., Balguri S.P., Avula B., Jablonski M.M., Majumdar S. (2019). In Situ Gel of Triamcinolone Acetonide-Loaded Solid Lipid Nanoparticles for Improved Topical Ocular Delivery: Tear Kinetics and Ocular Disposition Studies. Nanomaterials.

[25] Youssef A.A.A., Dudhipala N., Majumdar S. (2022). Dual Drug Loaded Lipid Nanocarrier Formulations for Topical Ocular Applications. IJN.

[26] Youssef A., Dudhipala N., Majumdar S. (2020). Ciprofloxacin Loaded Nanostructured Lipid Carriers Incorporated into In-Situ Gels to Improve Management of Bacterial Endophthalmitis. Pharmaceutics.

[27] Marathe S., Shadambikar G., Mehraj T., Sulochana S.P., Dudhipala N., Majumdar S. (2022). Development of α-Tocopherol Succinate-Based Nanostructured Lipid Carriers for Delivery of Paclitaxel. Pharmaceutics.

[28] Lallemand F., Daull P., Benita S., Buggage R., Garrigue J.-S. (2012). Successfully Improving Ocular Drug Delivery Using the Cationic Nanoemulsion, Novasorb. J. Drug Deliv..

[29] Singh M., Bharadwaj S., Lee K.E., Kang S.G. (2020). Therapeutic Nanoemulsions in Ophthalmic Drug Administration: Concept in Formulations and Characterization Techniques for Ocular Drug Delivery. J. Control. Release.

[30] Ammar H.O., Salama H.A., Ghorab M., Mahmoud A.A. (2009). Nanoemulsion as a Potential Ophthalmic Delivery System for Dorzolamide Hydrochloride. AAPS PharmSciTech.

[31] Naik J.B., Pardeshi S.R., Patil R.P., Patil P.B., Mujumdar A. (2020). Mucoadhesive Micro-/Nano Carriers in Ophthalmic Drug Delivery: An Overview. BioNanoScience.

[32] Abelson M.B., Udell I.J., Weston J.H. (1981). Normal Human Tear PH by Direct Measurement. Arch. Ophthalmol..

[33] Ban M.M., Chakote V.R., Dhembre G.N., Rajguru J.R., Joshi D.A. (2018). In-Situ Gel for Nasal Drug Delivery. Int. J. Dev. Res..

[34] Dhahir R.K., Al-Nima A.M., Yassir Al-bazzaz F. (2021). Nanoemulsions as Ophthalmic Drug Delivery Systems. Turk. J. Pharm. Sci..

[35] Lin L., Gu Y., Cui H. (2019). Moringa Oil/Chitosan Nanoparticles Embedded Gelatin Nanofibers for Food Packaging against Listeria Monocytogenes and Staphylococcus Aureus on Cheese. Food Packag. Shelf Life.

[36] Mitri K., Shegokar R., Gohla S., Anselmi C., Müller R.H. (2011). Lipid Nanocarriers for Dermal Delivery of Lutein: Preparation, Characterization, Stability and Performance. Int. J. Pharm..

[37] Uddin M.S., Mamun A.A., Kabir M.T., Setu J.R., Zaman S., Begum Y., Amran M.S. (2017). Quality Control Tests for Ophthalmic Pharmaceuticals: Pharmacopoeial Standards and Specifications. J. Adv. Med. Pharm. Sci..

[38] Rai V.K., Mishra N., Yadav K.S., Yadav N.P. (2018). Nanoemulsion as Pharmaceutical Carrier for Dermal and Transdermal Drug Delivery: Formulation Development, Stability Issues, Basic Considerations and Applications. J. Control. Release.

[39] Komaiko J.S., McClements D.J. (2016). Formation of Food-Grade Nanoemulsions Using Low-Energy Preparation Methods: A Review of Available Methods. Compr. Rev. Food Sci. Food Saf..

[40] Mishra N., Yadav K.S., Rai V.K., Yadav N.P. (2017). Polysaccharide Encrusted Multilayered Nano-Colloidal System of Andrographolide for Improved Hepatoprotection. AAPS PharmSciTech.

[41] Santos J., Calero N., Trujillo-Cayado L.A., Martín-Piñero M.J., Muñoz J. (2020). Processing and Formulation Optimization of Mandarin Essential Oil-Loaded Emulsions Developed by Microfluidization. Materials.

[42] Griffin W.C. (1954). Calculation of HLB Values of Non-Ionic Surfactants. J. Soc. Cosmet. Chem..

[43] Kiel J.W., Kopczynski C.C. (2015). Effect of AR-13324 on episcleral venous pressure in Dutch belted rabbits. J. Ocul. Pharmacol. Ther..

[44] Dong Y.-R., Huang S.-W., Cui J.-Z., Yoshitomi T. (2018). Effects of Brinzolamide on Rabbit Ocular Blood Flow in Vivo and Ex Vivo. Int. J. Ophthalmol..

[45] Rao H.L., Senthil S., Garudadri C.S. (2014). Contralateral Intraocular Pressure Lowering Effect of Prostaglandin Analogues. Indian J. Ophthalmol..

[46] Piltz J., Gross R., Shin D.H., Beiser J.A., Dorr D.A., Kass M.A., Gordon M.O. (2000). Contralateral Effect of Topical β-Adrenergic Antagonists in Initial One-Eyed Trials in the Ocular Hypertension Treatment Study. Am. J. Ophthalmol..

[47] Dunham C.N., Spaide R.F., Dunham G. (1994). The Contralateral Reduction of Intraocular Pressure by Timolol. Br. J. Ophthalmol..

[48] Arfaee F., Armin A. (2021). A Comparison between the Effect of Topical Tafluprost and Latanoprost on Intraocular Pressure in Healthy Male Guinea Pigs. J. Exot. Pet Med..

[49] Aihara M., Lindsey J.D., Weinreb R.N. (2002). Reduction of Intraocular Pressure in Mouse Eyes Treated with Latanoprost. Investig. Ophthalmol. Vis. Sci..

[50] Ota T., Murata H., Sugimoto E., Aihara M., Araie M. (2005). Prostaglandin Analogues and Mouse Intraocular Pressure: Effects of Tafluprost, Latanoprost, Travoprost, and Unoprostone, Considering 24-Hour Variation. Investig. Ophthalmol. Vis. Sci..

[51] Weinreb R.N., Lindsey J.D. (2005). The Importance of Models in Glaucoma Research. J. Glaucoma.

[52] Kim C.Y., Kuehn M.H., Anderson M.G., Kwon Y.H. (2007). Intraocular Pressure Measurement in Mice: A Comparison between Goldmann and Rebound Tonometry. Eye.

